# The Effect of Hydrogels with Different Chemical Compositions on the Behavior of Alkali-Activated Slag Pastes

**DOI:** 10.3390/gels8110731

**Published:** 2022-11-10

**Authors:** Joshua Prabahar, Babak Vafaei, Ali Ghahremaninezhad

**Affiliations:** Department of Civil and Architectural Engineering, University of Miami, Coral Gables, FL 33146, USA

**Keywords:** AAS, hydrogels, absorption

## Abstract

The effect of in-house synthesized hydrogels with different chemical compositions on the properties of alkali-activated slag pastes was examined. It was found that the teabag test and modified teabag test as a direct method and the flow test as an indirect method showed a similar trend in hydrogel absorption; however, the absorption values differ noticeably between the direct and indirect methods. The alkali-activated slag pastes with hydrogels demonstrated a significant reduction in autogenous shrinkage compared to the pastes without hydrogels. The creation of macrovoids by the hydrogels and change in pore structure resulted in a decrease in compressive strength and electrical resistivity of the pastes with hydrogels. The absorption and desorption of hydrogels in the pastes were tracked using X-ray microcomputed tomography (micro-CT), and it was shown that the onset of hydrogel desorption approximately coincided with the final setting time of the pastes.

## 1. Introduction

There have been rising concerns regarding the negative impacts that Portland cement production has on the environment. It is estimated that the energy consumption for Portland cement production is about 4–5 GJ per ton of Portland cement [1,2] and constitutes 5% of the energy usage in the industrial sectors worldwide [3]. Approximately 5–8% of the anthropogenic greenhouse gases released into the environment is attributed to Portland cement production. Therefore, there is an urgent need to find alternative binder materials with a lower carbon footprint and environmental impacts. Alkali-activated slag (AAS) binders have been studied as a green binder material with 40% lower embodied energy compared to the Portland cement binder [4]. AAS binders are obtained from ground granulated blast-furnace slag, which is a by-product of the steel making process [5]. Prior investigations have shown the promising advantages of AAS binders compared to the Portland cement binders, including better mechanical properties and improved chemical and fire resistance [6,7]. While AAS binders have demonstrated beneficial characteristics, issues related to volumetric changes have hampered their widespread utilization in the construction industry [8,9,10]. Previous research has evidenced larger autogenous shrinkage [8,9,10] and drying shrinkage [8,11,12,13,14,15,16] in AAS binders compared to Portland cement binders. Autogenous shrinkage in AAS binders results from development of the capillary forces in the microstructure when water is consumed and menisci are formed in the capillary pores [17].

In order to mitigate the effect of autogenous shrinkage in AAS binders, which could cause cracking, the use of shrinkage-reducing admixtures has been investigated [11,18]. Another approach to reduce autogenous shrinkage has been internal curing, in which water is provided from a reservoir dispersed in the interior of the materials. As reaction continues and water is consumed, the capillary forces provide the driving force for the release of water from these reservoirs into the surrounding microstructure to compensate for the loss of relative humidity [19]. Superabsorbent polymers (SAP) [19,20,21,22,23,24,25] and saturated lightweight aggregate [26,27,28,29] with a large water absorption capacity to serve as internal curing reservoirs have been studied in Portland cement binders. When the SAP comes into contact with water, the polymer networks of the SAP deprotonate and become negatively charged, resulting in the generation of repulsion within the polymer networks and swelling of the SAP [30]. When cations are present in the solution, the absorption of SAP is decreased due to the screening effect and complex formation [31].

The effect of SAP on autogenous shrinkage [19,20,21,22,23,24,25,32], mechanical properties [23,33,34,35,36], transport behaviors [20,21], microstructure [23,37,38,39,40], and hydration [21,39,41] of the Portland cement binders have been extensively investigated in the past. However, only a few studies have been focused on the use of SAP in AAS binders. Li et al. [42] showed that use of SAP reduced the drop in relative humidity and mitigated autogenous shrinkage resulting from self-desiccation. Oh and Choi [43] showed the beneficial effect of SAP in reducing autogenous shrinkage in AAS. Decreasing the drop in relative humidity and reducing autogenous shrinkage in AAS with SAP compared to AAS without SAP were noted in a prior study by Song et al. [44]. The use of SAP was found to reduce autogenous shrinkage in alkali-activated fly-ash-slag binders [45].

This paper aims to investigate the effect of SAP with different chemical compositions on their absorption in the AAS environment and on the autogenous shrinkage and other properties of AAS. To the best of the authors’ knowledge, such a study has not yet been conducted and will aid in filling a knowledge gap in the available literature.

## 2. Results and Discussion

### 2.1. Absorption in Solution

**Figure 1:**
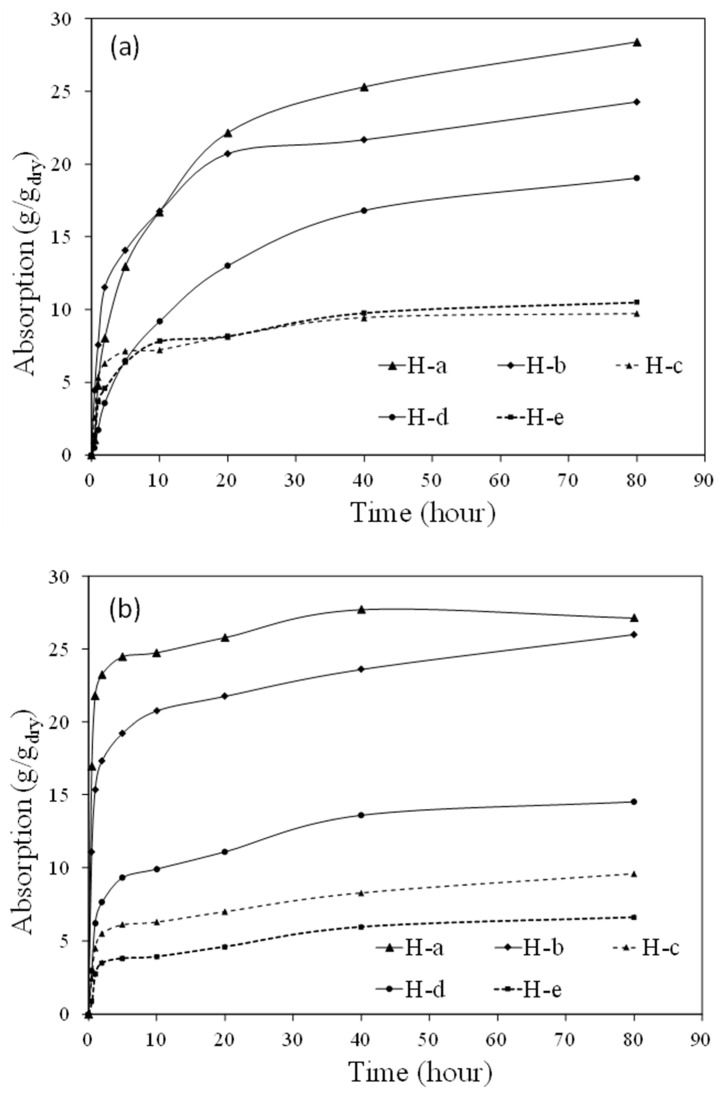
Absorption of different hydrogels in (**a**) activator solution and (**b**) slag and activator solution mixture.

The absorption results of the different hydrogels in the activator solution are shown in Figure 1a. It is seen that H-a demonstrated the highest absorption at 80 min, followed by H-b and H-d, and H-e and H-c showed the lowest absorption. It is noted that H-a and H-b exhibited relatively similar absorption in the first 10 min, but after this time the absorption of H-a increased more than that of H-b. The higher absorption of H-a can be attributed to the higher concentration of acrylic acid monomers (AA) in this hydrogel. At high pH of the activator solution (more than 13.5), AA monomers are completely deprotonated and acquire negative charges, leading to increased repulsive forces in the polymeric network of the hydrogel, which leads to swelling and higher absorption of the hydrogel [30,46].

### 2.2. Absorption in Slag Mixture

The absorption of the different hydrogels in a mixture of slag and activator solution is demonstrated in Figure 1b. Overall, the absorption of the hydrogels in the mixture followed a similar trend to that in the activator solution. It is noted that while the absorption of H-a was higher in the beginning, the two hydrogels acquired a similar absorption value at 80 min, as seen in Figure 1b. It is also seen that the absorption rate of H-a, H-b, and H-d appeared to be different in the mixture compared to in the solution. This could be due to the fact that in the slag and solution mixture, hydrogels might have less access to solution compared to the case of the hydrogels in the solution, where the hydrogels have access to an infinite amount of solution. It is worth noting that ions are released into the solution as a result of slag particle dissolution when it comes into contact with the activator solution. This can change the chemistry of the solution; however, it seems that the absorption of hydrogels was affected by the characteristics of the activator, and the release of ions did not significantly change the absorption behavior of the hydrogels. Ca^2+^ in solution has been shown to significantly reduce the absorption behavior of hydrogels containing AA [47,48,49]; since Ca^2+^ concentration in the slag system is very low compared to Portland cement systems [50,51], the absorption of H-a did not seem to be significantly affected in mixture of slag and activator solutions.

The lower absorption of H-e is attributed to the lower concentration of polymer phase in this hydrogel (66.7% polymer, 33.3% nanosilica), which reduces its absorption. Since H-c was prepared with an ionic solution, the difference in chemical potential between inside and outside of the hydrogel is reduced, which translates to a smaller thermodynamic force for absorption. In addition, the presence of ions in the hydrogel reduces absorption through the screening effect as well as complex formation between certain ions and anionic groups in the polymer network of the hydrogel [30,52,53,54].

### 2.3. Flow Test

The absorption results obtained from the flow test are shown in Table 1. It is interesting to observe that these absorption results follow a similar order to the absorption results measured by the modified teabag test as discussed previously. However, the absorption values vary between the two measurement methods. The absorption values obtained from the flow test are higher than those measured in the modified teabag test. It should be noted that the absorption values obtained from the flow test are more likely dependent on the amount of hydrogel used and the base solution/solid used in the mixture. A recent round robin study examined the absorption of hydrogels in cement pore solutions using the teabag method and filtration method. This study demonstrated the strengths and weaknesses of each method [55]. Optical imaging [36,56] and laser light diffraction [57] have been exploited in the absorption measurement of hydrogels. However, the absorption of hydrogels in the AAS systems has not been given adequate attention thus far, and more studies are needed to establish reliable methods of hydrogel absorption in alkali-activated slag systems.

### 2.4. Setting Time

The initial and final setting times of the pastes with different hydrogels are listed in Table 2. It is seen that the pastes with hydrogels showed a relatively shorter initial setting time, but their final setting time appeared to be increased compared to that of the paste without hydrogels, except in the case of the paste with H-d, which experienced a shorter final setting time. A prior investigation also demonstrated that AAS activated with NaOH and containing biochar as an internal curing agent had a longer final setting time compared to AAS without biochar with the same water/slag [58]. A possible reason for this behavior could be the lower availability of Na^+^ in the pastes with hydrogels, as a portion of the activator solution is taken up by the hydrogels and is not readily accessible to the slag for reaction. In addition, the different interactions between the hydrogels and the surrounding slag mixtures could affect the percolation of reaction products and formation of solid skeleton in the paste.

### 2.5. Heat Flow

**Figure 2:**
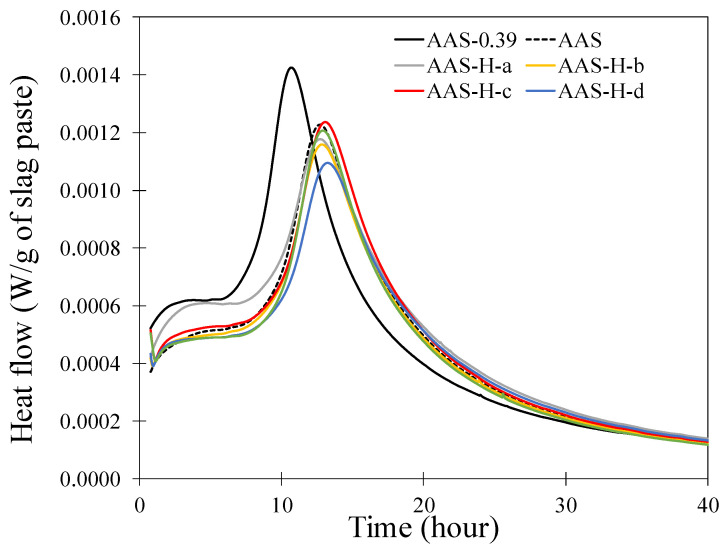
Heat flow of the pastes with and without hydrogels.

The heat flow of the reaction corresponding to the pastes with and without hydrogels is shown in Figure 2. It is noted that the main peak of AAS-0.39 occurred at 10–11 h, while the peaks of the other pastes with a water/slag of 0.44 occurred at 12.5–13.5 h. This main peak corresponds to the formation of the main reaction product, calcium-aluminate-silicate-hydrate (C-A-S-H) gel and the continued release of ions from slag [42,59]. The decrease in the reaction rate after the main peak is noted in all pastes and attributed to the reduced dissolution rate of slag due to the continuous formation of reaction products on slag surface [42]. It is observed that the main peak in AAS-0.39 was larger and occurred earlier than that of the other pastes that had an overall water/slag of 0.44. In AAS-0.39, the interstitial space between the slag is smaller than in the pastes with a water/slag of 0.44; thus, the number of ions in the pore solution in AAS-0.39 is smaller, and fewer Ca and Al ions are needed to be dissolved from slag into the solution to reach the critical supersaturation levels for Ca/Si and Al/Si [42]. Thus, AAS-0.39 reached its main peak earlier than the other pastes with water/slag of 0.44.

It is seen that the paste with hydrogels experienced a slightly delayed and smaller peak compared to the paste with the same water/slag but without hydrogel (AAS). In the pastes with hydrogels, a portion of the pore solution is absorbed by the hydrogels and is not immediately available for the reaction. The presence of the hydrogels and the uptake of the pore solution and its gradual release affect the interstitial space in the paste in such a way that is stipulated to slightly delay the reaction rate. Since the total solution in the pastes with different hydrogels was the same, the variation in the time and magnitude of the main peak observed in their heat flow responses could be attributed to their absorption/desorption characteristics and their influence on the interstitial space in the paste. However, a definite correlation between the reaction rate of the paste and hydrogel characteristics cannot be established from the data.

### 2.6. Autogenous Shrinkage

**Figure 3:**
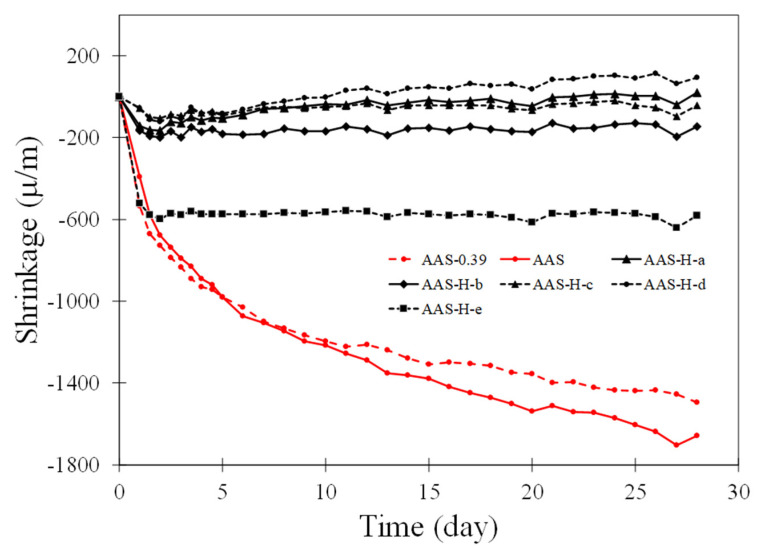
Autogenous shrinkage of the pastes with and without hydrogels.

The autogenous shrinkage of the pastes without hydrogels and pastes with hydrogels is depicted in Figure 3. It is observed that the autogenous shrinkage of the pastes without hydrogel showed similar behavior until day 11, at which point AAS-0.39 started to show slightly higher shrinkage compared to AAS. Of interest is the positive effect of the hydrogels in reducing autogenous shrinkage in the pastes. It is seen that the pastes with hydrogels, except for the paste with H-e, had autogenous shrinkage less than 200 μm/m; a small expansion can be seen in the case of the paste with H-d. The paste with H-e experienced a sharp increase in shrinkage within the first day, but then its autogenous shrinkage remained unchanged until day 28. While a direct relationship between the autogenous shrinkage of the pastes with hydrogel and the hydrogel absorption cannot be observed, it is interesting to note that H-e, which had the lowest absorption, exhibited the smallest reduction in autogenous shrinkage among the pastes with hydrogels. It should be noted that the hydrogel absorption and distribution as well as the kinetics of water release from hydrogels are the factors affecting the autogenous shrinkage of the pastes. The precise contributions of these factors are not fully understood, and more studies are needed to obtain a better understanding of the individual and synergistic effects of these parameters on autogenous shrinkage.

### 2.7. Compressive Strength

**Figure 4:**
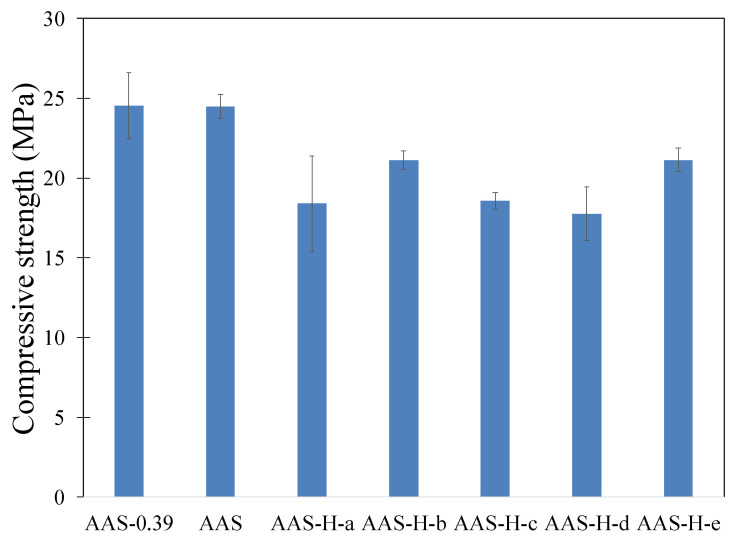
Compressive strength of the pastes with and without hydrogels after 28 days of curing.

The compressive strength after 28 days of the pastes with and without hydrogels is shown in Figure 4. It is seen that the pastes with hydrogels exhibited a lower compressive strength compared to the paste without hydrogels. This is primarily due to the presence of macrovoids created as a result of hydrogel swelling in the pastes. The effect of these macrovoids on reducing compressive strength has also been observed in previous investigations [42,60]. A comparison of the compressive strength results with the hydrogel absorption results shown in Figure 1a,b might suggest that the reduction in compressive strength appears to generally increase with hydrogel absorption, except in the case of AAS-H-b. As discussed in Section 4.1.2, the amount of dry hydrogels added was different for each hydrogel, with the aim to maintain a constant effective water/slag in the pastes with hydrogels. This indicates that the total porosity of the macrovoids was expected to be relatively similar in the pastes with hydrogels. However, the pastes with hydrogel with high absorption were expected to have fewer macrovoids compared to the pastes with low absorption. The difference in the compressive strength of the pastes with hydrogels could be due to the specific size distribution and total number of the macrovoids in the pastes [61].

### 2.8. Electrical Resistivity

**Figure 5:**
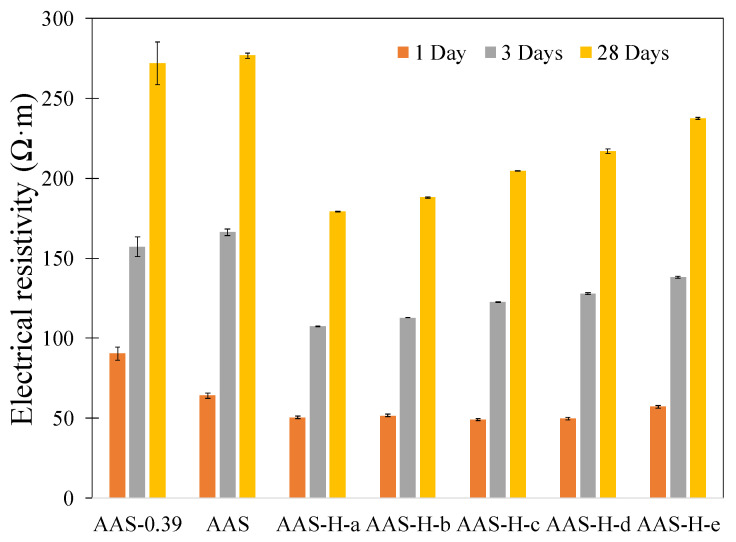
Electrical resistivity of the pastes with and without hydrogels at different ages of curing.

The electrical resistivity of the pastes with and without hydrogels at different ages is presented in Figure 5. It is observed that all pastes exhibited an increase in electrical resistivity with age, as the microstructure becomes more refined as the reaction continues and more reaction products are formed. It is noted that the pastes with hydrogels exhibited lower electrical resistivity compared to the pastes without hydrogels. It is interesting to observe that the reduction in electrical resistivity appeared to be generally related to the hydrogel absorption as shown in Figure 1a,b. It is postulated that the change in the capillary pore structure as well as in the number and size distribution of the macrovoids created by the hydrogels played an important role in affecting the electrical resistivity of the pastes with hydrogels. However, more studies are needed to establish a more definitive correlation between electrical resistivity and hydrogel absorption in the alkali-activated slag pastes containing hydrogels.

### 2.9. Thermogravimetric Analysis (TGA)

**Figure 6:**
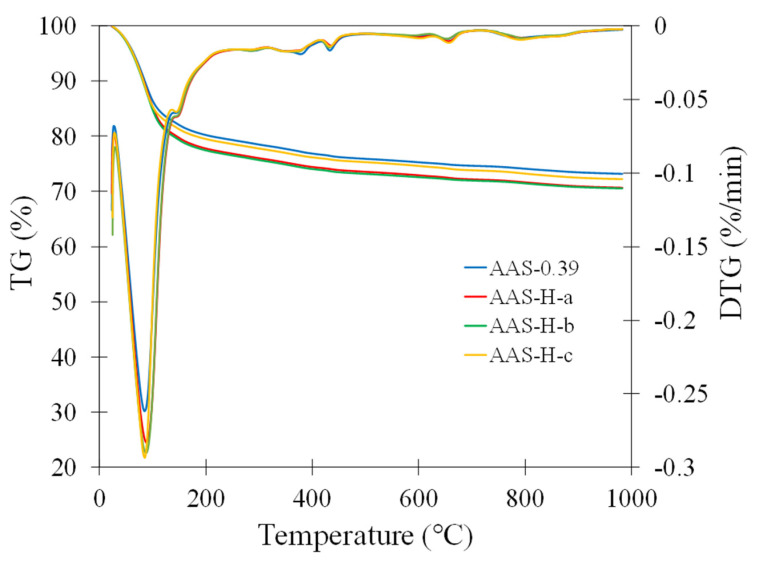
TGA and DTG curves of the paste without hydrogel and the pastes with select hydrogels after 28 days of curing.

The TG and differential TG (DTG) curves of the paste without hydrogel and the pastes with H-a, H-b, and H-c after 28 days of curing are shown in Figure 6. The pastes with these hydrogels were selected in the TGA analysis as these hydrogels showed distinct absorption behaviors, as shown in Figure 1a,b. The mass loss in the temperature range less than 105 °C is typically attributed to the evaporable water. The peak in the DTG curves at around 90 °C corresponds to the release of bound water in C-A-S-H. There are also peaks in the DTG curves at around 150 °C and 380 °C, which are attributed to the decomposition of hydrotalcite [62,63]. There is also a peak in the DTG curves at around 650 °C, which is due to the decomposition of carbonate. An accurate quantitative analysis of these phases in the microstructure using TGA is difficult due to the uncertainty regarding the signature decomposition temperature ranges of these phases. It is noted from Figure 6 that all TG curves appeared to have similar features, indicating that all pastes have similar phases and that no new phases were found. Thus, the addition of the hydrogels to the pastes did not significantly alter the chemical characteristics of the pastes.

### 2.10. Fourier Transform Infrared Spectroscopy (FTIR)

**Figure 7:**
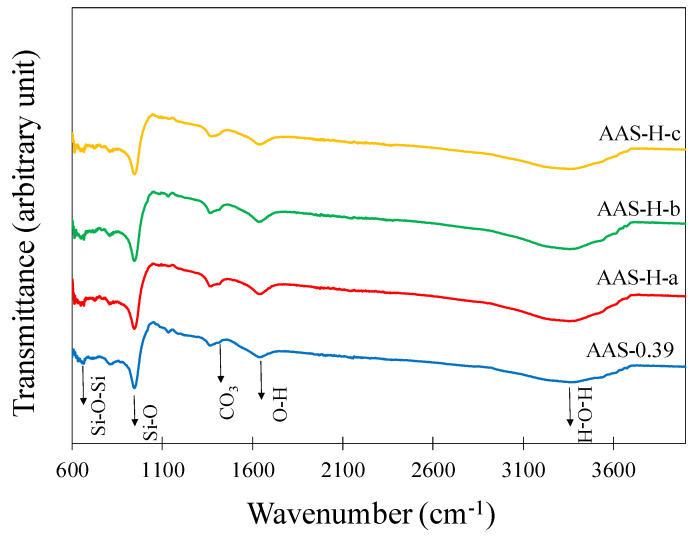
FTIR spectra of the paste without hydrogel and the pastes with select hydrogels after 28 days of curing.

The FTIR spectra of the paste without hydrogels and the pastes with select hydrogels after 28 days of curing are presented in Figure 7. The broad peak at around 3400 cm^−1^ and the peak at 1646 cm^−1^ are attributed to O-H stretching and H-O-H bending, respectively, in absorbed water [64]. The peaks at about 1410 cm^−1^ and 1360 cm^−1^ correspond to carbonate (C-O) [65]. A dominant peak at 946 cm^−1^ is observed in all spectra and is a characteristic of Si-O stretching, and the band at 660 cm^−1^ is attributed to Si-O-Si bending vibrations [64]. Consistent with the results obtained from the TGA analysis, it is seen that the FTIR spectra demonstrated similar characteristics. This leads to the conclusion that the addition of hydrogels in the pastes did not affect the chemical composition of the paste.

### 2.11. Micro-CT

**Figure 8:**
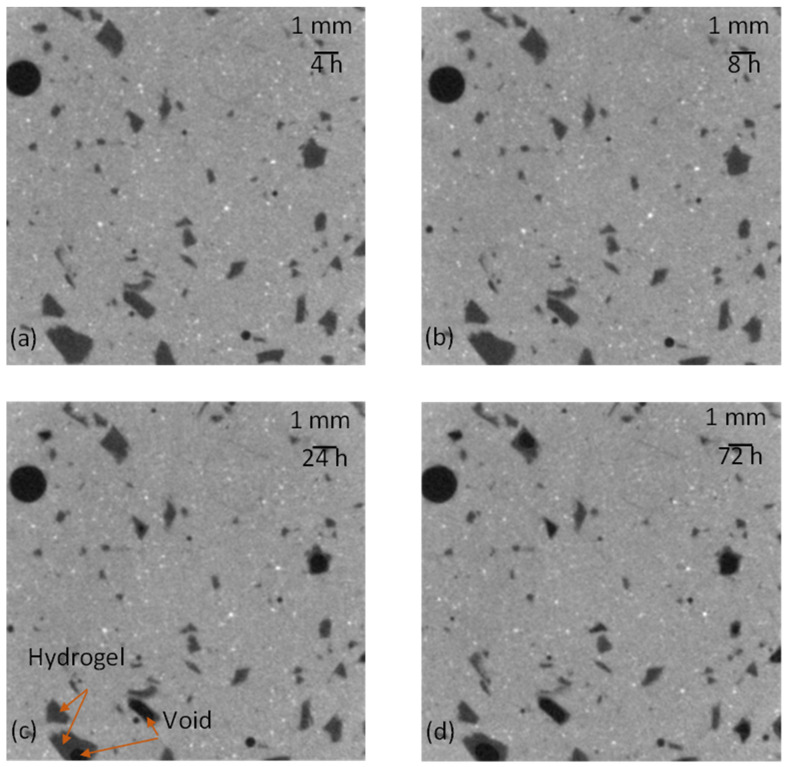
Two-dimensional images of a cross section of AAS-H-a at (**a**) 4 h, (**b**) 8 h, (**c**) 24 h, and (**d**) 72 h obtained from micro-CT.

**Figure 9:**
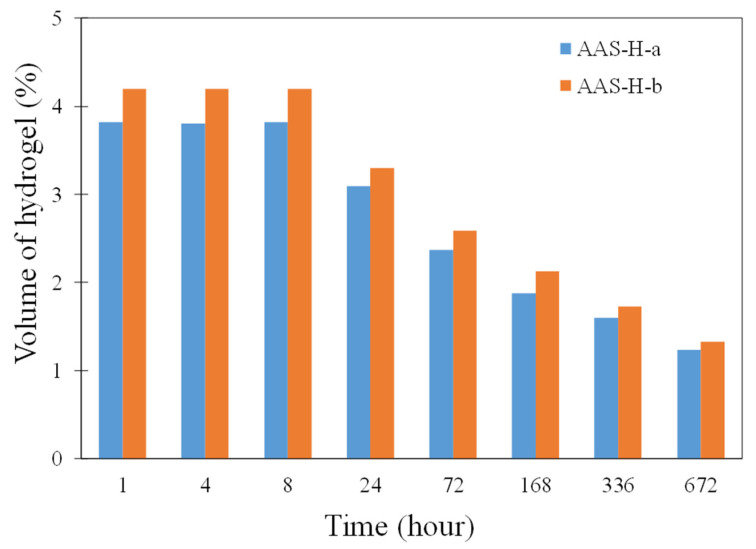
Total volume fraction of hydrogels in AAS-H-a and AAS-H-b at different times of curing.

**Figure 10:**
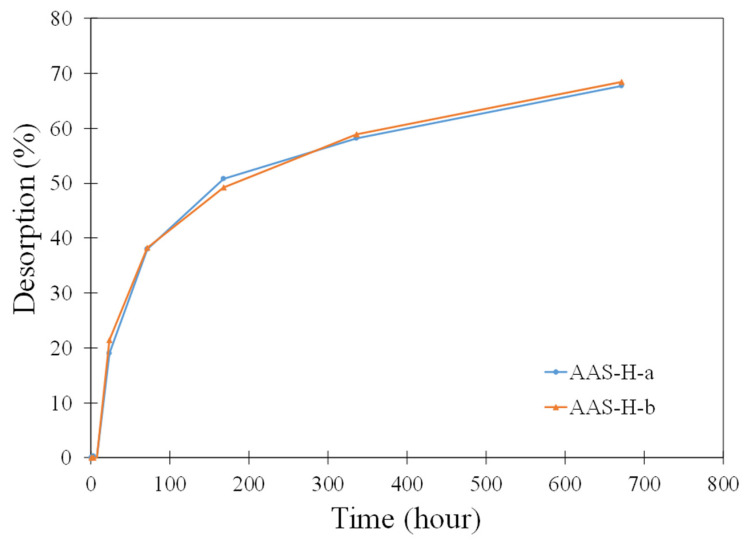
Desorption of hydrogels in AAS-H-a and AAS-H-b at different times of curing.

The 2D representations of a section of AAS-H-a at different times obtained by micro-CT are shown in Figure 8. The greyscale allows identification of the different phases in the microstructure of the paste. The white features are unreacted slag particles, dark grey features are absorbed hydrogels, and dark features are air voids or voids resulting from desorption of hydrogels. These features are marked in Figure 8c. The change in the total hydrogel volume fraction with time of AAS-H-a and AAS-H-b is shown in Figure 9. No significant change in the total volume fraction of the hydrogel in the first 8 h of the hydration is observed. There is a noticeable reduction in the total volume fraction of the hydrogels after 8 h, and the rate of reduction becomes smaller with time. The desorption of H-a and H-b as a function of time is depicted in Figure 10. The desorption at a time t was determined as (V_8h_ − V_t_)/V_in_, where V_t_ and V_8h_ are the total volume fraction of hydrogels at time t and time 8 h, respectively.

The setting times of the pastes with H-a and H-b are shown in Table 2. The desorption of hydrogels is driven primarily by the capillary forces in the microstructure. At the final setting time and the start of percolation of the solid skeleton in the paste, the capillary forces begin to develop, driving the release of solution from the hydrogel, which reduces the volume of the hydrogel. Prior studies [66,67,68] also showed that the start of hydrogel desorption coincided with the final setting time in Portland cement mixtures. Thus, in both AAS and Portland cement systems, the capillary forces are the main contributor to the release of water from the hydrogels.

**Figure 11:**
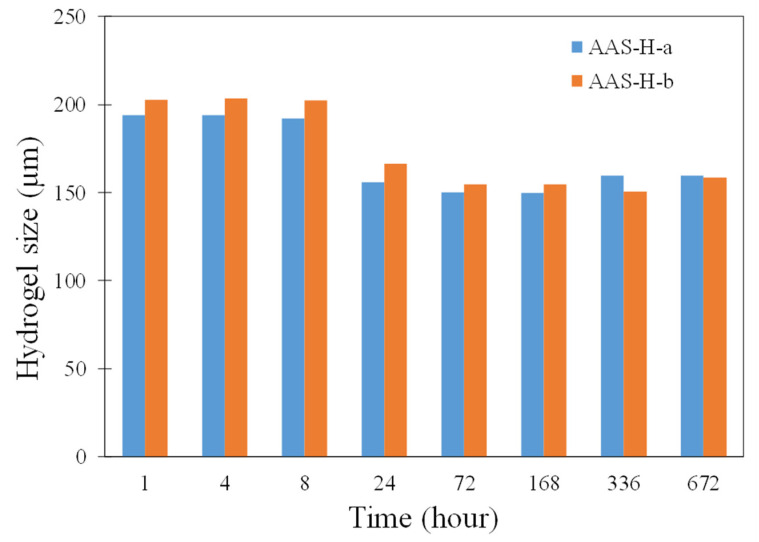
Size of hydrogels in AAS-H-a and AAS-H-b at different times of curing.

The hydrogel size as a function of time of H-a and H-b in the pastes is demonstrated in Figure 11. It is noted that H-b seemed to show a slightly higher absorption compared to H-a. It is also seen from the absorption results shown in Figure 1b that H-b gradually continued to increase in absorption for about one hour and would potentially surpass H-a after one hour. However, as discussed earlier, the absorption results obtained from the flow test indicated a slightly higher absorption of H-a compared to H-b; it should be borne in mind that the results obtained from the flow test correspond to the early age, approximately the first 10–20 min of the reaction. Our results indicate the need to investigate appropriate methods for accurate determination of hydrogel absorption in alkali-activated slag systems.

## 3. Conclusions

The behavior of AAS pastes containing hydrogels with different chemical compositions was investigated in this paper. The following conclusions are drawn from the findings of this study:

- The absorption of hydrogels in AAS was measured using the teabag test, modified teabag test, and flow test as a direct and an indirect method, respectively. While a similar hydrogel absorption trend was observed in the teabag test, modified teabag test, and flow test, the values of hydrogel absorption were largely different between the modified teabag test and flow test.
- It was shown that AAS with hydrogels showed a significant reduction in autogenous shrinkage compared to AAS without hydrogels. The ability of hydrogels to mitigate autogenous shrinkage in AAS systems is consistent with prior studies using Portland cement systems [19,20,21,22,23,24,25].
- Compressive strength and electrical resistivity of AAS with hydrogels were shown to decrease compared to AAS without hydrogels; the creation of macrovoids resulting from hydrogel absorption/desorption and change in capillary pore structure are stipulated to be the reasons for this reduction.
- TGA and FTIR indicated that the addition of hydrogels did not significantly affect the chemical characteristics of AAS.
- Micro-CT allowed for the monitoring of the absorption and desorption of hydrogels in AAS starting from the first few hours until several days after mixing. For the pastes studied in micro-CT, the onset of hydrogel desorption seemed to be close to the final setting time of the pastes. This observation is in agreement with prior investigations using Portland cement systems [66,67,68].
- It is recommended that the use of the flow test to determine hydrogel absorption in the alkali-activated slag systems be undertaken with caution. Further research is needed to explore the dependence of this method on various parameters, including the water/slag and the time from the first contact of the slag with the activator solution until the test is conducted.

## 4. Materials and Methods

### 4.1. Materials

#### 4.1.1. Hydrogels

**Figure 12:**
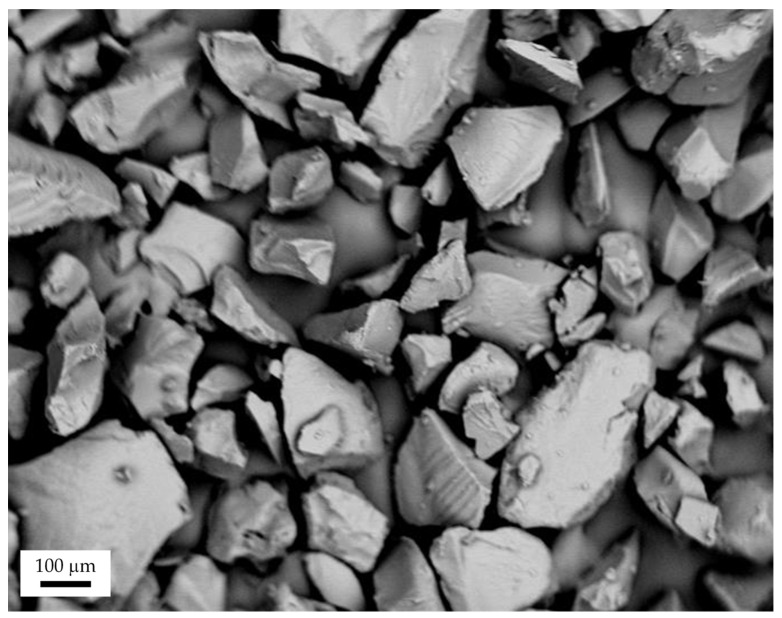
SEM image showing the particle size and morphology of H-d.

SAP used in construction applications is typically composed of hydrogels comprising a crosslinked copolymer of acrylamide and the salt of acrylic acid. In this study, five hydrogels with different chemical compositions were synthesized to vary their absorption properties. The chemical compositions of the hydrogels are listed in Table 3. All chemicals were procured from commercial vendors and utilized as received. The synthesis of the hydrogels was conducted using free radical polymerization as described in our previous publications [69,70] and is briefly detailed here. Acrylamide (AM) monomers and the crosslinker agent N,N’-methylenebisacrylamide (MBA) were dissolved in distilled water and stirred vigorously. For the hydrogel containing acrylic acid monomers (AA), AA was first neutralized with sodium hydroxide (NaOH), and then AM and MBA were added. For hydrogels containing nanosilica (NSi) or alginate (Alg), AM and MBA were added to previously stirred solution of NSi or Alg. After degassing the solution with argon for about 3 min, ammonium persulfate (APS) was added to serve as the polymerization initiator. Then, the solution was transferred into a beaker and placed in an oven to gel at a temperature of 50 °C for 3 h. Then, the hydrogels were taken out of the beakers and submerged into distilled water to remove the monomers that remained unreacted. In order to allow ionic species to alter the molecular structure of the hydrogel, the hydrogel containing Alg was submerged in an ionic solution instead of water. The ionic solution was prepared following the formulation described in Tunstall et al. 2017 [71]. Then, the hydrogels were removed from the distilled water, broken into smaller pieces, and dried in an oven at 80 °C. Dried hydrogels were ground into powder with a coffee grinder and then sieved to obtain hydrogel particle sizes in the range of 75–425 µm. A scanning electron microscopy image of the hydrogels is shown in Figure 12. It can be seen that the hydrogel particles had an angular shape.

#### 4.1.2. Paste Mix Design

The chemical composition of the ground granulated blast-furnace slag, referred to as slag, used in AAS pastes is provided in Table 4. The mix designs and the designations of the AAS pastes with and without hydrogel are shown in Table 5. Pastes without hydrogels were prepared with a water/slag of 0.39 and 0.44. All pastes with hydrogel were mixed with a water/slag of 0.44. The concentration of the hydrogels in the pastes with hydrogels was determined so as to reach the same effective water/slag of 0.39, and the amount of solution absorbed in the hydrogels was the same in all pastes (water/slag = 0.05). To this end, the absorption values of hydrogels used were obtained from the average absorption in the first 20 min in the modified teabag test as described in Section 4.2. The paste without hydrogels with a water/slag of 0.39 was used for comparison purposes. The alkaline activator used was a solution of 1.78 M sodium hydroxide (NaOH). For the paste with the water/slag of 0.39, the concentration of NaOH had to be slightly adjusted to 2 M in order to maintain the targeted water/slag. A ZYLA superplasticizer at a concentration of 0.17% per slag mass was used to improve the workability of the mixtures.

All slag pastes with hydrogels were first dry mixed in a bucket for 5 min. The activator solution was then poured into the dry mixture and allowed to rest for 30 s to permit the solution to percolate throughout. The paste was then mixed with a mixer at a slow speed for 15 s followed immediately by a high speed for 45 s. The mixing was stopped for 30 s to allow time to scrape the interior wall of the mixing bucket. The mixing was then resumed at a high speed for 60 s. The pastes were poured into 50 mm cubic molds and compacted on a vibrating table for 30 s. The molds were sealed using a plastic foil to prevent loss of moisture from the paste at room temperature. After 24 h the paste cubes were demolded and stored in double layers of plastic foil stored in a shoebox container at room temperature until testing.

### 4.2. Absorption Using Teabag Test and Modified Teabag Test

Determining the absorption of internal curing agents in AAS binders is necessary for accurate design of the material. A common method to measure the absorption of internal curing agents in solution is the teabag test [30,52]. However, there are limitations associated with this testing methodology: namely, solution adsorption to the surface of internal curing agents and solution entrapment in interstitial space between internal curing agent particles. Additionally, since the teabag test is performed in a solution medium, the potential effect of the reacting binder particle on the absorption of the internal curing agents is neglected. To resolve this limitation, a modified teabag test was conducted to account for the interactions between the reacting slag particles and the hydrogel. This more accurately simulates absorption of the hydrogel under more realistic conditions. First, 2 g of hydrogel and 10 g of slag were dry mixed for 2 min and poured into the teabags. The teabags were submerged in separate beakers containing the activator solutions, and the solutions were sealed using a Parafilm foil. At different times, the teabags were removed from the solution and their mass measured using an analytical balance with a 0.001 g resolution. The surface of the teabags was dried to exclude surface adsorbed solution in the mass measurement. Since the solution can also be absorbed by the teabag and dry slag, the test was performed with a teabag containing 10 g dry slag without hydrogel. The following equation was employed to determine hydrogel absorption:

(1) $$\mathrm{Absorption}=\frac{M_{w}{-M}_{s}{-M}_{d}}{M_{d}}$$

where $M_{d}$, $M_{s}$, and $M_{w}$ are the mass of dry hydrogel, teabag with slag only, and teabag with hydrogel and slag, respectively. Teabag tests were also done to obtain the absorption of hydrogel alone in the activator solution. Triplicate samples were used in each test.

### 4.3. Absorption Using Flow Test

A flow test in accordance with ASTM C1437 was conducted as an indirect method to measure the hydrogel absorption in the paste. This indirect way of measuring hydrogel absorption based on flowability has been utilized in previous studies [33,60,72]. Paste was filled in a cone with a height of 5 cm, bottom diameter of 10 cm, and top diameter of 7 cm and allowed to sit for 5 min on a table. Then, the cone was removed, and the table was dropped 25 times in a time span of 15 s. The two perpendicular diameters of the paste were measured, averaged, and reported as the flow value of the paste. The paste without hydrogel had the water/slag of 0.39; in the paste with hydrogel the hydrogel/slag was 0.3% for all hydrogels. Since hydrogel uptakes water, additional solution in incremental steps was added to the paste with hydrogels until the same flow value as for the paste without hydrogel was achieved. The additional solution added to the paste with hydrogel was assumed to be absorbed by the hydrogels and used to determine hydrogel absorption.

### 4.4. Setting Time Test

The Vicat test was employed to measure the initial and final setting times of the pastes. The penetration depth of the Vicat needle was measured every 30 min until initial setting time and every 15 min until final setting time. The measurements were conducted at five different locations on the paste, and the average is reported.

### 4.5. Autogenous Shrinkage Test

The autogenous shrinkage measurements of the pastes up to 28 days of curing were conducted in accordance with ASTM C1698-16. To this end, pastes were poured into corrugated polyethylene tubes (42 cm in length and 2.9 cm in diameter). The change in the length of the samples was tracked using a DC linear variable differential transformer (LVDT). The zero time of the measurement coincided with the final setting time of the pastes. Duplicate samples were employed for each paste, and the average autogenous shrinkage was calculated.

### 4.6. Isothermal Calorimetry Test

Isothermal calorimetry was employed to measure the heat flow of the pastes. Pastes in the amount of 7 g were mixed, placed into glass ampoules, compacted with a tamper, and sealed with a lid. The glass ampoules were loaded into a TA Instrument isothermal calorimeter, which was preconditioned at 23 °C. The heat flow and the cumulative heat of each paste were measured for 100 h.

### 4.7. Thermogravimetric Analysis (TGA)

In order to gain insight into the chemical phases in the paste, TGA was performed on the paste without hydrogel and the pastes with select hydrogels after 28 days of curing. Broken pieces of cubes used in the compressive strength test were placed in acetone for 24 h. Then, they were ground into fine powder and sieved using a sieve #60. Approximately 30 mg of the powder passing through the sieve was poured into glass vials and placed in a vacuum oven for 24 h at 50 °C. The TGA analysis was conducted using a Shimadzu DTG-60H up to a temperature of 1000 °C and with a temperature rate of 20 °C/min.

### 4.8. Fourier Transform Infrared Spectroscopy (FTIR)

FTIR was conducted on the pastes after 28 days to study the potential effect of hydrogels on the reaction products in the pastes. The sample preparation for this test was similar to that for TGA. FTIR scans were performed in the transmission mode in a Perkin Elmer Paragon 1000 FTIR with an attenuated total reflectance accessory. Samples were scanned at the resolution of 4 cm^−1^ and in the range of 600–4000 cm^−1^.

### 4.9. Micro-CT Analysis

In order to examine the hydrogel absorption and desorption in the pastes, the microstructure of the pastes with H-a and H-b was imaged using micro-CT. Pastes with H-a and H-b were prepared and poured into a small 2 mL Eppendorf tube, sealed, and loaded into a Skyscan 1273 (Bruker). The samples were scanned at different times of 1 h, 4 h, 8 h, 24 h, 72 h, 168 h, 336 h, and 672 h after the paste was mixed to monitor the change in the volume of the hydrogel in the microstructure as the reaction continued. The samples were scanned with a resolution of 12 mm/pixel, rotational step of 0.6 deg, voltage of 100 kV, current of 150 mA, and X-ray exposure time of 635 ms. Due to the small size of the samples, the scanning was completed in about 60 min. The software CTAnalyzer 1.20.8 (Bruker) was employed for 3D reconstruction.

### 4.10. Compressive Strength Test

In order to assess the effect of hydrogels on the compressive strength of the pastes, paste cubes at an age of 28 days were utilized. The cubes were tested in a SATEC machine. Three cubes were employed for each paste.

### 4.11. Electrical Resistivity Test

The electrical resistivity of the paste cubes with and without hydrogels at the age of 1, 3, and 28 days was measured utilizing the Electrochemical Impedance Spectroscopy (EIS) method [73,74]. A Gamry Reference 600 potentiostat/galvanostat was used for the EIS measurement. The measurement was conducted with a frequency range of 10^6^–10 Hz and an amplitude of 250 mV. The following equation was used to determine the electrical resistivity of the pastes.

*ρ =* (*r*)(*a*)/(*t*) (2)

where *r, t*, and *a* are the measured resistance of the cubes, cube thickness, and cube side surface area, respectively.

## Figures and Tables

**Table 1:** Absorption of the different hydrogels obtained from the flow test.

Hydrogel	Absorption (g/g_dry_)
H-a	73
H-b	47
H-c	33
H-d	36
H-e	10

**Table 2:** Initial and final setting times of the pastes with and without hydrogels.

Paste	Initial Setting Time (min)	Final Setting Time (min)
AAS-0.39	136	356
AAS	132	438
AAS-H-a	114	468
AAS-H-b	125	533
AAS-H-c	117	503
AAS-H-d	108	409
AAS-H-e	102	497

**Table 3:** Chemical compositions of the hydrogels synthesized in this study.

Hydrogel	AM (g)	AA (g)	MBA (g)	APS (g)	NaOH (g)	Alg (g)	NSi (g)	Distilled Water (g)
H-a	10	10	0.05	0.128	1.35	-	-	100
H-b	18	2	0.05	0.128	0.27	-	-	100
H-c	20	-	0.05	0.128	-	0.6	-	100
H-d	20	-	0.05	0.64	-	-	-	100
H-e	20	-	0.05	0.64	-	-	10	100

**Table 4:** Oxide composition of the slag used in this study.

Composition	(%)
SiO_2_	31.6
Al_2_O_3_	11.0
Fe_2_O_3_	0.9
CaO	44.6
MgO	6.4
Na_2_O	0.2
K_2_O	0.4
SO_3_	3.1
LOI	1.8

**Table 5:** Mix designs of the AAS pastes with different hydrogels.

Designation	Overall Water/Slag	Effective Water/Slag	Superplasticizer (% per Slag Mass)	Hydrogel (% per Slag Mass)	Water (g)	NaOH (g)
AAS-0.39	0.39	0.39	0.17	-	1170	93.6
AAS	0.44	0.39	0.17	-	1320	93.6
AAS-H-a	0.44	0.39	0.17	0.17	1320	93.6
AAS-H-b	0.44	0.39	0.17	0.19	1320	93.6
AAS-H-c	0.44	0.39	0.17	0.45	1320	93.6
AAS-H-d	0.44	0.39	0.17	0.31	1320	93.6
AAS-H-e	0.44	0.39	0.17	0.56	1320	93.6

## Data Availability

Not applicable.

## Abbreviations

Alkali-activated slag (AAS); Acrylamide (AM); Acrylic acid (AA); N,N’-methylenebisacrylamide (MBA); nanosilica (NSi); ammonium persulfate (APS); alginate (Alg).
